# Exploration of Anti-infectives From Mangrove-Derived *Micromonospora* sp. RMA46 to Combat *Vibrio cholerae* Pathogenesis

**DOI:** 10.3389/fmicb.2020.01393

**Published:** 2020-07-10

**Authors:** Hema Bhagavathi Sarveswari, Shanthini Kalimuthu, Karthi Shanmugam, Prasanna Neelakantan, Adline Princy Solomon

**Affiliations:** ^1^Quorum Sensing Laboratory, Centre for Research in Infectious Diseases, School of Chemical and Biotechnology, SASTRA Deemed to be University, Thanjavur, India; ^2^Faculty of Dentistry, The University of Hong Kong, Pok Fu Lam, Hong Kong

**Keywords:** anti-infectives, anti-virulence, anti-biofilm, rare marine Actinobacteria, *Micromonospora* species, marine compounds, *Vibrio cholerae*

## Abstract

*Vibrio cholerae*, the etiological agent of cholera, employs quorum sensing (QS) pathways to control the expression of virulence factors, including the production of cholera toxin and biofilm formation. Acquired antibiotic resistance in *V. cholerae* draws attention to the development of novel therapeutics that counteract virulence, rather than the viability of the pathogen. In this context, we explored the anti-infective potential of rare marine Actinobacteria (RMA) from a mangrove ecosystem. Here, we report the effects of *Micromonospora* sp. RMA46 against *V. cholerae in vitro*. The RMA46 organic extract was non-bactericidal to *V. cholerae* cells and non-cytotoxic to macrophage RAW264.7 cell lines. RMA46 inhibited the formation of *V. cholerae* biofilms and downregulated the QS global switches LuxO and HapR, as well as other virulence genes including *ct*, *tcp*, and *hap*A. *In silico* molecular docking simulation of RMA46 ethyl acetate extract with LuxO and HapR revealed that 2-methoxy-4-vinylphenol and hexahydro-3-(phenylmethyl)-pyrrolo[1,2-*a*]pyrazine-1,4-dione could interact with the active sites of LuxO and HapR and potentially inhibit them. This study highlights *Micromonospora* sp. RMA46 as a potential source of anti-infectives against *V. cholerae*.

## Introduction

*Vibrio cholerae*, a Gram-negative bacterium from the family Vibrionaceae, causes cholera, a self-limiting acute diarrheal disease (Zhu and Mekalanos, 2003). This contagious disease spreads primarily through the fecal–oral route and by consumption of contaminated food and water (Sack et al., 2004; Clemens et al., 2017). *V. cholerae* is classified into more than 200 serogroups on the basis of their lipopolysaccharide antigens, yet only two serogroups, O1 and O139, are associated with epidemic outbreaks of cholera (Wang et al., 2016). It is estimated that about 2.86 million people are affected by cholera in endemic countries, resulting in 95,000 deaths annually (Ali et al., 2015).

Antibiotic therapy is crucial for the treatment of cholera, as it abates the period of diarrhea, the amount of rehydration fluids, and the density of vibrios excreted in the stools (World Health Organization [WHO], 2019). Over the years, the rapid emergence of extensive drug-resistant (XDR) and multidrug-resistant (MDR) *V. cholerae* strains possess a great public health threat, owing to their association with treatment failure and potential dissemination of resistance among other pathogens through mobile genetic elements (MGEs) (Chowdhury et al., 2016; Clemens et al., 2017; Verma et al., 2019; Gladkikh et al., 2020). The above caveats compel the need for developing novel strategies to thwart *V. cholerae* infection. Numerous studies have focused on developing compounds that target other fitness tactics of bacteria instead of developing newer antibiotics (Hema et al., 2016, 2017).

*V. cholerae* demonstrates extraordinary fitness by coordinated group behavior in expressing virulence factors through quorum sensing (QS) pathways (Miller and Mekalanos, 1984; Hammer and Bassler, 2003). There are two major parallel QS systems in *V. cholerae* comprising cholera autoinducer-1 (CAI-1), autoinducer 2 (AI-2), and their corresponding cognate inner membrane receptors (CqsS and LuxP/Q) (Miller et al., 2002; Rutherford et al., 2011). These individual receptors converge the information to a global response regulator, LuxO, in response to the low and high concentrations of their respective signals. Under low signal strength (LSS), the receptors function as kinases and phosphorylate LuxO, which activates the transcription of quorum regulatory RNAs (Qrrl-4) and controls the translation of two master regulators, AphA and HapR. The *qrr*1-4 small RNAs upregulate the expression of AphA and concurrently repress the expression of HapR (Rutherford et al., 2011). In turn, AphA regulates the expression of biofilm activator (VpsT), toxin-coregulated pilus (TCP), and cholera toxin (CTX), commencing the process of adhesion and production of CTX in humans (Lenz et al., 2004; Yang et al., 2010; Rutherford et al., 2011). These processes are key to the colonization of this bacterium in the intestine and form microcolonies. By contrast, under high signal strength (HSS), the receptors function as phosphatases, leading to dephosphorylation and inactivation of LuxO. This results in the downregulation of virulence and biofilm formation by the expression of hemagglutinin/protease A (HapA). HapA expression facilitates the dissemination of bacteria from the intestinal cells. Thus, LSS promotes infection, whereas HSS promotes bacterial detachment from the intestinal cells, resulting in dissemination (Zhu et al., 2002; Hammer and Bassler, 2003).

Anti-virulence therapy using synthetic and natural molecules that interfere with QS pathways in pathogens has been explored extensively. These molecules function either as an inhibitor or an agonist of the factors involved in QS signaling, affecting their biosynthesis, detection, signal transduction, and even as enzymes that inactivated the QS signal molecules (Defoirdt, 2018). Such QS molecules that occur naturally have been sourced from various plants, microorganisms, and higher organisms from a wide range of terrestrial and marine ecosystems. Among microorganisms, the phylum Actinobacteria has been a prodigious source of anti-virulence agents, providing a range of chemicals that interfere with the QS system of various human pathogens (Sarveswari and Solomon, 2019). In this work, we isolated and screened 42 rare marine Actinobacteria (RMA) from mangrove soil against rugose *V. cholerae* HYR14 biofilm and virulence. The organic solvent of RMA displaying a significant anti-biofilm activity was also analyzed for its potential to inhibit virulence mechanism through gene expression studies. Finally, the potential interaction of the active principles identified in the organic extract of RMA with the *V. cholerae* global response regulators LuxO and HapR was analyzed through *in silico* docking studies.

## Materials and Methods

### Target Bacterial Strains and Culture Conditions

*Vibrio cholerae* biofilm-forming rugose strain, HYR14 obtained from NICED, India, was used throughout this study (Chowdhury et al., 2016). The strain was cultured in thiosulfate–citrate–bile salts–sucrose (TCBS) agar to confirm cell viability and propagated under standard growth conditions in Luria–Bertani (LB) broth at 37°C. An inoculum size of 0.2 (OD_595_) ≈ 10^5^ cells were used for all the experiments (Hema et al., 2016).

### Soil Sampling From Mangrove Ecosystem

Mangrove soil was sampled from Muthupet Mangrove Reserve, Tamil Nadu, India (Supplementary Table S1). Soil samples were collected using sterile containers from a site that was 20 m away from the shore and with 20 m depth. The samples were transported to the laboratory under cooling conditions and stored at 4°C in a dry condition prior to examination (Hong et al., 2009). The physio-chemical parameters such as salinity and temperature were measured using a handheld refractometer (Erma, Japan) during sample collection. The pH of the samples was determined using Eutech pH meter (Oakton, United States).

### Pretreatment of Mangrove Soil and Selective Isolation of Rare Marine Actinobacteria

The soil was sieved through a large mesh to eliminate organic particles and was homogenized using a mortar and pestle. About 1 g of the homogenized samples was pretreated by 120°C for 60 min (dry heat) (Pisano et al., 1986) and by 1.5% of phenol for 30 min at 30°C (Pisano et al., 1986) and through moist heating with sterilized mangrove water at 50°C for 15 min (Takahashi, 2004). The pretreated soil samples were then serially diluted [1:10 (v/v) ratio] with sterile saline solution, and about 100 μl was spread plated on various isolation media. For selective isolation of RMA, International Streptomyces Project medium (ISP1 to ISP7) (Shirling and Gottlieb, 1966), actinomycetes isolation agar, starch casein agar, and humic acid–vitamin agar (Hayakawa and Nonomura, 1987) (HiMedia Laboratories, Mumbai, India) (Supplementary Table S2) supplemented with the filter-sterilized cycloheximide (40 μg/ml) and nalidixic acid (40 μg/ml) were used. The inoculated Petri plates were then incubated at 28°C for 4–6 weeks and regularly checked for the growth of actinomycetes-like colonies. Single discrete actinomycetes-like colonies grown on spread plated isolation media were re-subcultured on to ISP4 and actinomycetes isolation agar and further incubated at 28°C for the isolation of pure colonies (Shirling and Gottlieb, 1966). Collection of pure colonies of the RMA isolates was stored at 4°C on ISP4 agar slants and also as lyophilized cultures for short-term and long-term storages, respectively.

### Cultural, Morphological, and Biochemical Characterization of Rare Marine Actinobacteria Isolates

The RMA isolates collected from various isolation media were subjected to morphological characterization and biochemical analysis (Pridham and Gottlieb, 1948; Shirling and Gottlieb, 1966). Light microscopy was used to observe the morphologies of Actinobacteria isolates including the arrangement of mycelium and formation of spores by Gram’s staining. The culture characteristics, including growth patterns on different media, pigmentation, colony color, presence, and color of aerial and substrate mycelium on various ISP media (ISP1 to ISP7), were determined.

### Fermentation and Ethyl Acetate Extract Preparation

International Streptomyces Project medium no. 2 (ISP2) (0.5 g of peptone, 0.3 g of malt extract, 0.3 g of yeast extract, and 1.0 g of dextrose pH 6.2 ± 0.2) was used as fermentation medium for the exploration of the secondary metabolites produced by the 42 mangrove Actinobacteria isolates. The ISP2 medium was sterilized by autoclave at 121°C at 15 lb for 20 min. Initially, about four to five colonies from pure culture were inoculated into modified ISP2 media supplemented with 2.0 g/l of calcium carbonate in an Erlenmeyer conical flask and incubated at 150 rpm at 28°C for 7 days. After the incubation period, 5% (v/v) of well-grown culture from the calcium carbonate-supplemented ISP2 medium was inoculated into ISP2 media in an Erlenmeyer conical flask. The flask was incubated at 150 rpm at 28°C for 14 days. The uninoculated ISP2 medium served as a medium control, to ensure purity. After fermentation, the spent medium of the isolates was centrifuged at 5,000 rpm for 10 min, and the culture supernatant was filtered through a 0.2 μm nitrocellulose filter. The cell-free extracts were stored at -20°C. For the extraction of secondary metabolites by ethyl acetate, the cell-free filtrate was extracted twice with an equal volume of ethyl acetate, and the organic extract was condensed by evaporating ethyl acetate using a rotational evaporator (Heidolph Laborata, 4001) (Balasubramanian et al., 2017). The organic extract was resuspended in ethyl acetate and was used for further studies.

### Efficacy of Rare Marine Actinobacteria Cell-Free Extract Against *Vibrio cholerae* HYR14 Biofilm

The antibiofilm activity of various dilutions of the 42 RMA cell-free extracts (50, 25, 12.50, 6.25, and 3.13%) was investigated. One hundred microliters (final volume) of LB broth containing various concentrations of RMA cell-free extract was aliquoted into a 96-well microtiter plate. Ten microliters of an overnight-grown culture of *V. cholerae* HYR14 strain that has been diluted up to 1:100 (OD_595_ = 0.2) was inoculated into each well, and the plates were incubated at 37°C for 24 h. Controls were included for all the experiments. After the incubation period, the plates were subjected to crystal violet staining. The medium and the planktonic cells present in the 96-well microtiter plates were aspirated at the end of the incubation period. The non-adherent HYR14 cells present in the wells were discarded by washing the wells thrice with sterile 1× phosphate-buffered saline. The microtiter plates were then air-dried for 20 min at ambient temperature to fix the adherent biofilms. To the dried wells, 100 μl of 0.2% (w/v) crystal violet was added and incubated for 20 min at room temperature. After the incubation period, the excess stain was washed off with sterile water twice, and the plates were air-dried completely. The bound crystal violet was solubilized by pipetting 100 μl of 33% glacial acetic acid into each well (Kaur et al., 2016). The absorbance of the wells was measured at 595 nm using an iMark^TM^ microplate absorbance reader (Bio-Rad Laboratories, Inc., United States).

### Nucleic Acid-Based Identification of Rare Marine Actinobacteria Strain Demonstrating *Vibrio cholerae* Biofilm Inhibitory Activity

The extraction of the genomic DNA of the RMA strain exhibiting a significant inhibitory activity against the *V. cholerae* HYR14 biofilm was performed using commercially available DNeasy Blood and Tissue kits (Qiagen, Germany) as per the manufacturer-recommended protocol (Ahmed et al., 2008). Until further use, the eluted DNA was stored at -20°C. Nested PCR targeting the 16SrRNA of the eubacterial genome was done using primers: forward primer (U1): 5′-TTGGAGAGTTTGATCCTGGCTC-3′ and reverse primer U4: 5′-GGACTACCAGGGTATCTA-3′ for the first round of nested PCR and forward primer (U2): 5′-TTGGAGAGTTTGATCCTGGCTC-3′) and reverse primer (U3): 5′-GCGGCTGGCACGTAGTTAG-3′ for the second round of nested PCR (Therese et al., 1998). The reaction conditions were set as described in Kharel (Sitaula) et al. (2018). The amplified product was then cyclo-sequenced, purified, and loaded to ABI3200 genetic analyzer (Applied Biosystems, United States) and sequenced as described previously (Aarthi et al., 2011). The obtained sequence was analyzed using BioEdit software (version 7.0.5.3) and was compared with 16S rRNA sequence database of National Center for Biotechnology Information (NCBI) using nucleotide BLAST (Altschul et al., 1990). The phylogenetic tree was constructed using the neighbor-joining method (Perrière and Gouy, 1996) and was validated using bootstrap analysis. The sequence was submitted to GenBank for accession number.

### Quantification of Biofilm Formation and Eradication

To evaluate the effect of RMA46 organic extract on mature *V. cholerae* biofilm, 100 μl of fresh LB broth was aliquoted into a 96-well microtiter plate. This was inoculated with 10 μl of an overnight-grown culture of *V. cholerae* HYR14 strain that had been diluted up to 1:100 (OD_595_ = 0.2). The microtiter plate was incubated at 37°C for 24 h. After the incubation period, the non-adherent planktonic cells were aspirated gently without disturbing the biofilm. To the wells, 100 μl (final volume) of LB broth containing various concentrations of RMA46 organic extract (50–0.13 mg/ml) was aliquoted. The microtiter plate was incubated again at 37°C for 24 h. Following the incubation period, crystal violet assay was performed to evaluate the potential of RMA46 organic extract on eradication of *V. cholerae* biofilm. Untreated and vehicle controls were also included for all experiments (Balasubramanian et al., 2017).

To quantify the effect of RMA46 organic extract on the formation of *V. cholerae* biofilm, 100 μl (final volume) of LB broth containing various concentrations of RMA46 organic extract (50–0.13 mg/ml) was aliquoted into 96-well microtiter plate. Ten microliters of an overnight-grown culture of *V. cholerae* HYR14 strain that has been diluted up to 1:100 (OD_595_ = 0.2) was inoculated into each well, and the plates were incubated at 37°C for 24 h. After the incubation period, the plates were subjected to crystal violet staining. Untreated and vehicle controls were also included for all experiments.

For crystal violet staining, the medium and the planktonic cells present in the 96-well microtiter plates were aspirated at the end of the incubation period. The non-adherent HYR14 cells present in the wells were discarded by washing the wells thrice with sterile 1 × phosphate-buffered saline. The microtiter plates were then air-dried for 20 min at ambient temperature to fix the adherent biofilms. To the dried wells, 100 μl of 0.2% (w/v) crystal violet was added and incubated for 20 min at room temperature. After the incubation period, the excess stain was washed off with sterile water twice, and the plates were air-dried completely. The bound crystal violet was solubilized by pipetting 100 μl of 33% glacial acetic acid into each well (Kaur et al., 2016). The absorbance of the wells was measured at 595 nm using an iMark^TM^ microplate absorbance reader (Bio-Rad Laboratories, Inc., United States).

### Confocal Laser Scanning Microscopic Analysis

*V. cholerae* HYR14 (OD_595_ = 0.1) and RMA46 ethyl acetate extract at a concentration of (9.3 mg/ml) were inoculated into LB medium in cell culture plate containing sterile borosilicate coverslips and were incubated statically at 37°C for 24 h. After 24 h, the suspension was aspirated and removed carefully. The biofilms established on the coverslips were rinsed with 0.9% NaCl solution. Stock solutions of SYTO^TM^ 9 green fluorescent stain (Thermo Fisher Scientific) and propidium iodide red fluorescent dyes (BacLight, Invitrogen Ltd.) were prepared at 1:1 ratio and stored at −20°C until further use. Briefly, 200 μl of the dyes was added to the coverslips and incubated at room temperature for 15 min under dark conditions. After incubation, the dyes were aspirated, and the coverslips were carefully washed with 0.9% NaCl. The washed coverslips were air-dried at ambient temperature and fixed on a sterile glass slide. These slides were observed under a 40× objective using a confocal laser scanning microscope (Olympus FLUOVIEW, FV1000). The biomass, average thickness, and roughness coefficient of biofilms were analyzed using COMSTAT package in MATLAB R2017b (Heydorn et al., 2000).

### Cell Viability Assay

The effect of the RMA46 ethyl acetate extract on host cell viability was assayed using murine macrophages RAW264.7, cultured in Dulbecco’s modified Eagle’s medium (DMEM) supplemented with 10% fetal bovine serum (FBS), 1% penicillin/streptomycin (w/v), and 1% L-glutamine using the MTT (3-(4,5-dimethylthiazol-2-yl)-2,5-diphenyltetrazolium bromide) assay. About 1 × 10^4^ RAW264.7 cells/well were seeded in a 96-well microtiter plate and incubated at 37°C in a CO_2_ incubator for 24 h. After the incubation period, the cells were treated with various concentrations (0.13, 0.39, and 9.375 mg/ml) of *Micromonospora* sp. RMA46 ethyl acetate extract and incubated for another 24 h at 37°C in a CO_2_ incubator. After 24 h, 1 mg/ml of MTT was added, and the microtiter plate was incubated at 37°C for 3 h. Dimethyl sulfoxide (DMSO) was added to dissolve the formazan crystals, and absorbance was read at 570 nm using a microplate reader spectrophotometer (Synergy H1) (Kartik et al., 2020).

### Extraction of RNA and Quantitative Analysis of Virulence Gene Expression in *Vibrio cholerae* HYR14

Total RNA was extracted from the RMA46 extract-treated and untreated *V. cholerae* HYR14 from stationary phase culture (24 h) using RNeasy^®^ Protect Bacteria Mini Kit (Qiagen), according to the manufacturer’s guidelines. The purity and integrity of the isolated RNA were checked using NanoDrop (Thermo Fisher Scientific, United States). Preparation of cDNA was performed using the iScript^TM^ cDNA Synthesis kit with a concentration of 100 ng following the manufacturer’s guidelines. The following conditions were set for cDNA synthesis; 24°C for 5 min (annealing), 42°C for 30 min (extension), and 85°C for 5 min (inactivation). The effect of RMA46 ethyl acetate extract on the virulence gene expression in the HYR14 strain was analyzed by qRT-PCR using primers listed in Supplementary Table S4. The 16s rRNA was used as the reference gene. The relative gene expression was calculated using the 2^–Δ^ ^Δ^ ^CT^ method (Vezzulli et al., 2015; Hema et al., 2017).

### Gas Chromatography–Mass Spectrometry Analysis

Separation and identification of the active compounds present in the ethyl acetate extract of RMA46 were performed with a PerkinElmer Clarus 500 GC-MS system. Helium was employed as a carrier gas at a flow rate of 1 ml/min, and the injector temperature was set at 280°C. The program of the column temperature was held at 50°C for 1 min and increased at 10°C/min to 150°C (1 min hold), to 250°C for 1 min hold, and then at 15°C/min to 300°C (3 min hold). The mass spectrometer was operated at a mass range of 40–450 amu. One microliter of RMA46 extract dissolved in ethyl acetate was injected into the system. The separated compounds were identified by comparing their spectra with those in the National Institute of Standards and Technology (NIST) mass spectral library (Ravichandiran et al., 2012).

### Computational Studies

Two-dimensional structures of the compounds identified in the *Micromonospora* sp. RMA46 ethyl acetate were drawn using MarvinSketch^1^. The ligands were prepared for molecular docking using LigPrep (Schrödinger, LLC, New York, NY, 2020). The primary structure of the ATP binding domain of LuxO (Q9KT84) was retrieved from Uniprot^2^. Protein BLAST (BLASTp) was performed for the query sequence against Protein Data Bank (PDB) to identify the structurally similar homolog. The QS signal integrator LuxO of *Photobacterium angustum* with the PDB accession number 5EP0 was chosen as the template. Homology modeling was performed using the protein structure prediction tool implemented in BioLuminate (Schrödinger, LLC, New York, NY, 2020). The modeled protein was subjected to a constrained energy minimization using GROMACS (Kutzner et al., 2019), and the quality of the modeled structure was further evaluated using the SAVES server^3^. The experimentally solved structure of HapR protein (2PBX) was downloaded and used for molecular docking simulations. Both the LuxO and HapR proteins were prepared using the protein preparation wizard (Sastry et al., 2013). A grid box was generated and was centered on the binding sites of the target proteins, and the docking simulation was performed using extra precision (XP) docking algorithm implemented in Glide (Schrödinger, LLC, New York, NY, 2020) (Friesner et al., 2006).

### Statistical Analysis

GraphPad Prism software version 8.0.2 (GraphPad Software Inc., San Diego, CA, United States) was used for analyzing the data statistically. The significance was analyzed by Student *t*-test with *p* set at *p* ≤ 0.05. All the assays were performed in triplicates, and the results have been expressed as mean ± SD.

## Results

### Mangrove Soil Is Rich in Morphologically Distinct Rare Marine Actinobacteria

Non-*Streptomyces* like colonies that were selectively subcultured for the isolation of pure RMAs produced a wide range of colonies from round convex to dry, raised, irregularly edged colonies. These colonies also produced varied colony pigments from white, yellow, pink, orange, brownish-orange, gray, and black. Out of the 42 isolates, only one isolate (RMA44) produced diffusible pigment. Light microscopy revealed that a variety of structures including coccus, rod-coccus, coryneform, and short- and long-branched sometimes filamented hyphal formations. Many of these hyphal-producing strains also formed oval spores. The pattern of hyphae and spore formation also varied significantly among RMA isolates. A few isolates changed colony pigmentation from orange to black as the colonies got older (Supplementary Table S3). This showed that the mangrove soil supported a rich diversity of Actinobacteria.

### Mangrove-Derived Rare Marine Actinobacteria Extract Could Attenuate *Vibrio cholerae* HYR14 Biofilm

**FIGURE 1:**
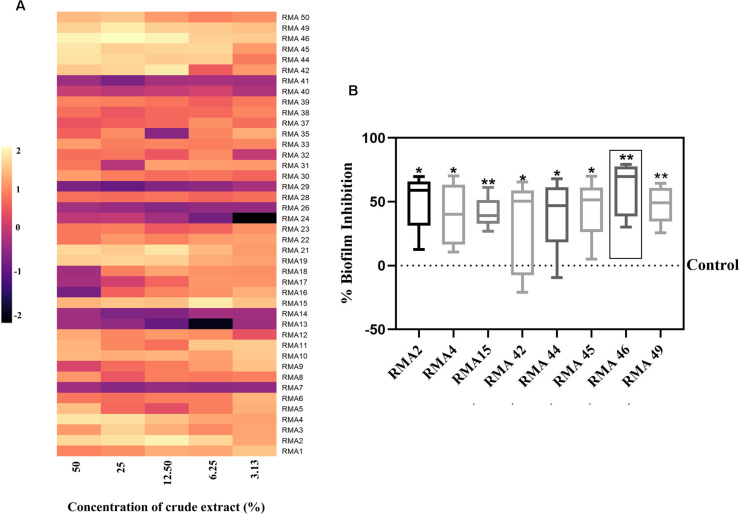
**(A)** Effect of various concentrations of cell-free extract of 42 rare marine Actinobacteria (RMA) [50, 25, 12.50, 6.25, and 3.13% (v/v)] on 24 h biofilm of *Vibrio cholerae* HYR14. **(B)** Comparison of various concentrations [50, 25, 12.50, 6.25, and 3.13% (v/v)] of cell-free extracts of selected RMA strains that demonstrated significant HYR14 biofilm inhibitory activity. RMA46 displays the maximum (*p* < 0.01) inhibition than do all the other strains. A one-sample *t*-test was used for significance analysis. *p < 0.05, and **p < 0.01.

The cell-free culture supernatants of all the 42 RMA isolates were screened for an anti-biofilm activity. The isolates RMA2, RMA4, RMA15, RMA42, RMA44, RMA45, RMA46, and RMA49 exhibited a > 50% biofilm inhibitory activity (Figure 1A). However, the cell-free culture supernatant of RMA46 exhibited a consistent level of ≥ 30% inhibition of HYR14 biofilm without a bactericidal activity at all concentrations (v/v) chosen in the study (Figure 1B and Supplementary Figure S1). Therefore, the strain RMA46 was chosen for further investigations.

### Isolate RMA46 Belongs to the Genus *Micromonospora*

The complete 16S rRNA sequence of the RMA46 strains (724 bp) determined in this study was deposited in GenBank database (accession number MK788184.1). To study the evolutionary relationship, similarity search of 16S rRNA sequence of RMA46 was performed against 16S rRNA database in National Center for Biotechnology Information (NCBI). The closely related sequences of RMA46 were used to construct the phylogenetic tree. The result showed that the strain RMA46 is closely related to *Micromonospora* sp. 171111 (accession number EF538723) and was clustered together in the phylogenetic tree (Supplementary Figure S2).

### *Micromonospora* sp. RMA46 Ethyl Acetate Extract Effectively Inhibits Biofilm Formation by HYR14 Than by Eradicating Preformed Biofilms

**FIGURE 2:**
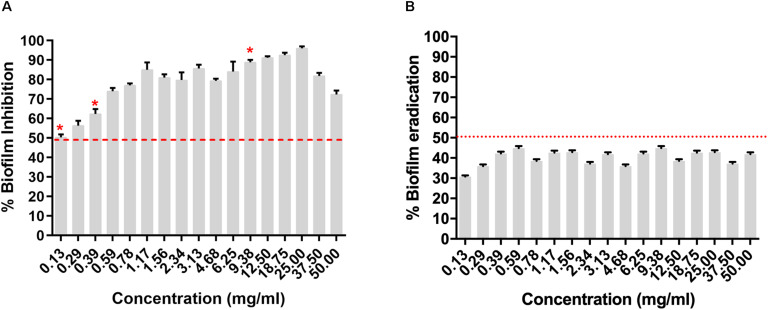
**(A)** Dose-dependent inhibition of *Micromonospora* sp. RMA46 organic extract on the formation of *Vibrio cholerae* HYR14 biofilm. **(B)** Dose-dependent effect of RMA46 organic extract on preformed biofilm *V. cholerae* HYR14 biofilm. * Effective concentrations chosen for further studies.

The RMA46 organic extract (50–0.13 mg/ml) was evaluated for its *Vibrio cholerae* HYR14 biofilm inhibitory/disruptive potential. The RMA46 ethyl acetate extract inhibited HYR14 biofilms ≥ 50% at low concentrations (0.39–0.13 mg/ml). At higher concentrations (0.59–50 mg/ml), the RMA46 ethyl acetate extract effectively inhibited > 70% of the HYR14 biofilm formation (Figure 2A). Overall, the data were consistent to show a dose-dependent increase in the inhibitory potential of RMA46 ethyl acetate extract against the *V. cholerae* HYR14 biofilm. On the contrary, the RMA46 ethyl acetate extract was unable to eradicate > 50% of preformed biofilm (Figure 2B). Because the biofilm inhibitory concentration (BIC) of RMA46 ethyl acetate extract ranges from 50 to 90%, three effective BICs were chosen – BIC_50_ (0.13 mg/ml), BIC_60_ (0.39 mg/ml), and BIC_90_ (9.38 mg/ml) – to validate anti-infective potential.

### RMA46 Ethyl Acetate Extract Affects the Thickness and Biomass of *Vibrio cholerae* Biofilm

**FIGURE 3:**
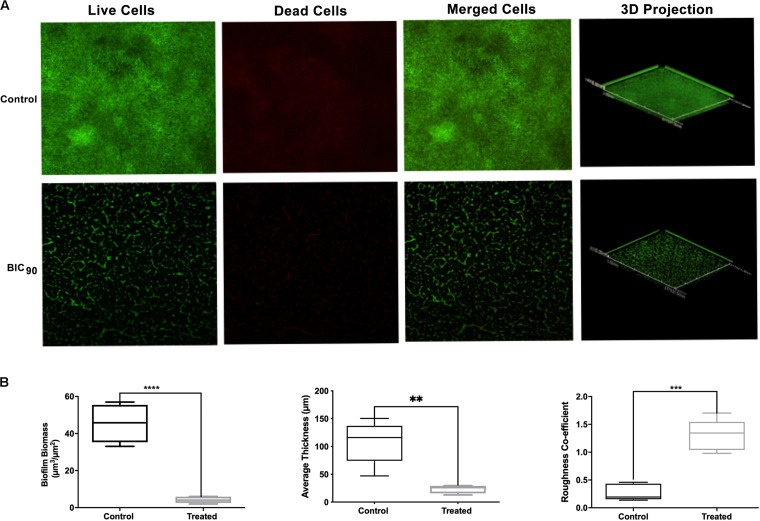
**(A)** Confocal laser scanning microscopy of HYR14 biofilm in the presence of the RMA46 extract, visualized by red and green fluorescent vital dyes. **(B)** COMSTAT analysis on the biomass, average thickness, and roughness coefficient of HYR14 biofilm treated with RMA46 organic extract. Student’s *t*-test was used for significance analyses. ^*^p < 0.05 was considered significant. **p < 0.01, ***p < 0.001, and ****p < 0.0001.

Investigation on the effect of RMA46 ethyl acetate extract on HYR14 biofilm formation on coverslips was performed using confocal laser microscopy and COMSTAT analysis. Treatment with BIC_90_ (9.38 mg/ml) revealed a detached biofilm matrix, whereas the untreated control sample showed highly condensed and compact biofilms (Figure 3A). COMSTAT analysis revealed that the untreated *V. cholerae* formed thicker biofilms (107.7 μm) with higher biomass (45.4 μm^3^/μm^2^) and a roughness coefficient of 0.27. Yet the RMA46 ethyl acetate treated HYR14 biofilms was 23.2 μm and 4.2 μm^3^/μm^2^ in thickness and biomass, respectively. The roughness coefficient of the RMA46 treated HYR14 biofilm is 1.30. These results signify that RMA46 ethyl acetate leads to an irregular distribution and reduced biomass of HYR14 than does the untreated HYR14 biofilm (Figure 3B).

### RMA46 Ethyl Acetate Extract Does Not Affect Mammalian Cell Viability

**FIGURE 4:**
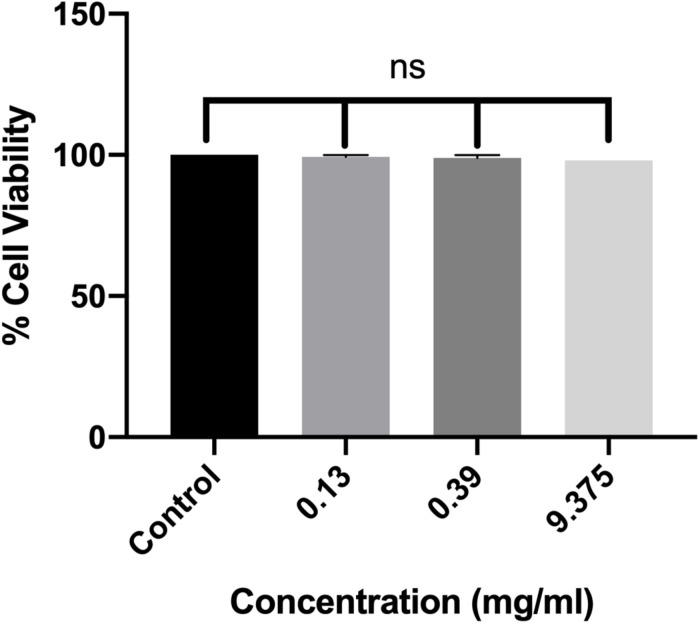
Dose-dependent effect of RMA46 ethyl acetate extract on the cell viability. Experiments were performed in triplicates, and difference in mean values was compared with that of the untreated experiment control. One-way ANOVA was used for multiple comparison analyses. ns denotes not significant.

The effect of ethyl acetate extract of RMA46 on the viability of host cells was assessed against macrophage RAW264.7 cell lines. Three concentrations, representing BIC_50_, BIC_60_, and BIC_90_ (0.13, 0.39, and 9.37 mg/ml, respectively), were chosen to investigate the extract’s lethality for 24 h. In all treatments compared with the control (untreated macrophage RAW264.7 cells), the viability of the cells was not affected, which indicated the non-toxic nature of the extract without affecting the cell viability (Figure 4).

### RMA46 Extract Interferes With HYR14 Molecular Machinery

**FIGURE 5:**
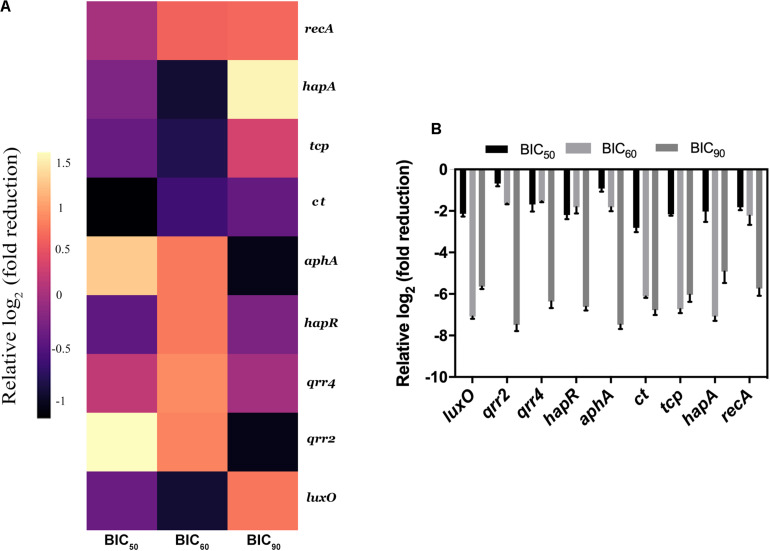
Expression analysis of *Vibrio cholerae* virulence genes by quantitative PCR. **(A)** Heat map of real-time qRT-PCR to show the effect of RMA46 organic extract on virulence gene expression in *V. cholerae*. **(B)** Comparison between the various inhibitory concentration of RMA46 extract on HYR14 virulence-associated genes.

The real-time analysis of the expression pattern of *V. cholerae* HYR14 global switches (LuxO and HapR) and its downstream virulence genes – *ct*, *tcp*, small regulatory RNAs (*qrr2* and *qrr4*), *aphA*, and *hapA* – were quantified differentially under RMA46 organic (ethyl acetate) extract induced/uninduced state. At LSS, the global switch, LuxO is switched on to regulate the transcription of many other vital genes including the *tcp* and *ct* to encode toxin coregulated pilus and CTX, respectively. The small regulatory rRNA termed as *qrr*1-4 along with Hfq (RNA chaperone) binds with mRNA transcripts of *hap*R. HapR repression leads to the formation of biofilm. Similarly, at HSS, the de-repression of HapR leads to the suppression of biofilm formation and upregulation of protease production, causing dissemination of *V. cholerae*. In this regard, the expression analysis at BIC_50_ showed a 2–3 log_2_ fold reduction in the expression of LuxO and HapR and its downstream virulence genes *hapA*, *tcp*, and *ct*. Similarly, the treated (BIC_60_) cells showed a > 2 log_2_ fold downregulation in the expression of the global regulators (LuxO and HapR) and virulence genes (*tcp* and *ct*). Overall, the data were significant to show that RMA46 extract interferes with the HYR14 molecular machinery in a dose-dependent manner to downregulate to > 2–5 log_2_ fold, which confirmed that the entire virulence mechanism of *V. cholerae* has been switched off (Figures 5A,B).

### Metabolic Profiling of RMA46 and Their Molecular Interaction With Active Sites of LuxO and HapR

With the use of gas chromatography–mass spectrometry (GC–MS), a total of 43 secondary metabolites were identified in the RMA46 extract belonging to different categories of secondary metabolites including alkanes (4.6%), alkenes (23.2%), carboxylic acids (25.5%), esters (6.9%), dairy ketone (4.6%), phenols (2.6%), and terpene (2.3%). The extract also contained aromatic monocyclic and heterocyclic compounds (20.9%) and other hydrocarbons (4.6%). Interestingly, the RMA46 extract also contained an oxaspiro compound (2.3%) (Table 1 and Supplementary Figure S3).

**TABLE 1:** List of compounds identified in *Micromonospora* sp. RMA46 ethyl acetate extract using GC–MS.

**S. no.**	**Class of compound**	**Name of the compound**	**Molecular weight (g/mol)**	**Retention time (min)**	**Peak area (%)**
1	Carboxylic acids	2-Methoxy-propanoic acid	104	4.26	9.51
		3-Methyl-butanoic acid	102	5.60	2.96
		2-Methyl-butanoic acid	102	5.74	1.58
		4-Methyl-3-pentenoic acid	114.14	7.54	0.19
		α-Isopropylidene-aminooxy-propionic acid	145.15	9.11	0.99
		Benzoic acid	122.12	12.38	0.90
		Bis(2-methylpropyl)ester 1,2-benzenedicarboxylic acid	278.34	31.11	0.99
		*N*-[2-[4-(Acetyloxy)-3-methoxyphenyl]ethyl]-acetamide	251	33.78	1.31
		*N*-(3-Methylbutyl)acetamide	129.2	10.73	0.29
		*N*-(2-Phenylethyl)acetamide	163	21.88	2.47
		Bis(2-methylpropyl)ester 1,2-benzenedicarboxylic acid	278.34	31.11	0.99
2	Alkenes	1-Dodecene	168.31	11.05	0.83
		1-Tetradecene	196	15.19	4.26
		1*H*-Indene, 1-methylene-	128.17	11.67	0.68
		1-Non-adecene	266.5	23.56	7.66
		Hexadecane	266	23.80	0.56
		(*E*)-5-Octadecene	252.48	29.30	0.56
		Cetene	224.43	29.45	9.03
		3- Methyl-, (*E*)-5-undecene	168	15.51	0.09
		(*E*)-3-Eicosene	280	32.93	7.02
		1-Docosene	308	35.79	5.53
3	Alkanes	Dodecane	170.33	11.18	0.40
		Tetradecane	198	15.35	0.97
4	Phenols	2,4-Di-*tert*-butylphenol	206	20.99	0.95
		2-Methoxy-4-vinylphenol	150.177	14.31	1.06
5	Esters	2-Phenylethyl ester acetic acid	164	12.62	0.12
		Diphenyl ether	170.2	16.37	0.04
		2-Ethylhexyl salicylate	250	30.07	0.16
6	Aromatic heterocyclic compounds	2-Coumaranone	134	12.76	0.45
		2,3-Dihydro-benzofuran	120	13.32	1.50
		Biphenyl	154	15.92	0.11
		Hexahydro-pyrrolo[1,2-*a*]pyrazine-1,4-dione	154.17	31.36	2.25
		Hexahydro-3-(2-methylpropyl)-pyrrolo[1,2-*a*]pyrazine-1,4-dione	210	33.44	12.99
		Hexahydro-3-(phenylmethyl)-pyrrolo[1,2-*a*]pyrazine-1,4-dione	244	42.51	3.15
7	Heterocyclic hydrocarbon	4,5-Dimethyl-1,3-dioxane	116.16	6.34	0.11
8	Terpene	4-(1-Methylethyl)-2-cyclohexen-1-one	138.20	7.41	0.03
9	Aromatic hydrocarbon	1,4-Dichlorobenzene	147	8.11	0.14
10	Cyclic ketones	3-Methyl-1,2-cyclopentanedione	112.13	8.71	0.15
11	Aromatic monocyclic hydrocarbons	Phenylethyl alcohol	122.16	10.44	0.32
		Benzeneacetic acid	136	14.02	11.58
		Tetrahydro-4-hydroxy-6-pentyl-2*H*-pyran-2-one	186.25	20.68	0.09
12	Ketone	Benzophenone	182.22	26.30	0.58
13	Oxaspiro compound	7,9-Di-*tert*-butyl-1-oxaspiro(4,5)deca-6,9-diene-2, 8-dione	276.4	32.17	2.09

*GC–MS, gas chromatography–mass spectrometry.*

**FIGURE 6:**
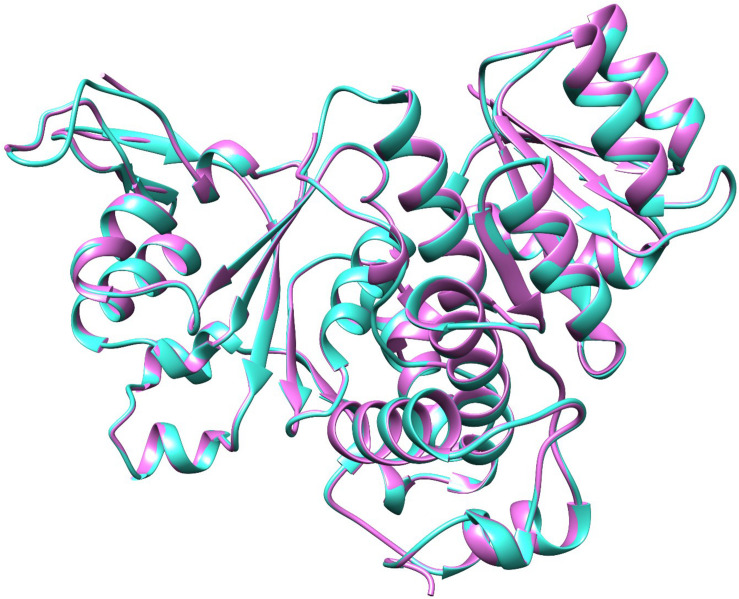
Molecular superimposition of LuxO of *Photobacterium angustum* (template) and *V. cholerae*. The LuxO of *P. angustum* is represented in purple color and the LuxO of *V. cholerae* in turquoise color.

**FIGURE 7:**
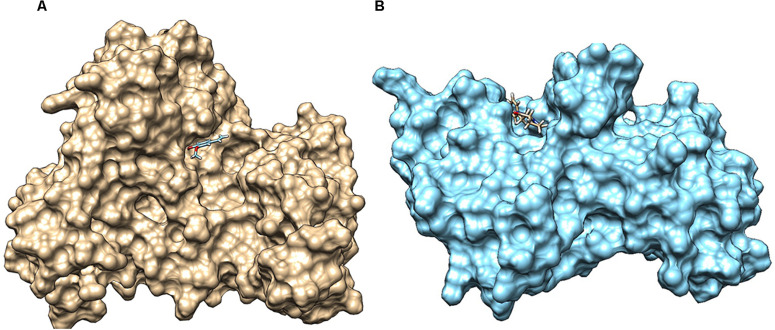
**(A)** Docked complex of 2-methoxy-4-vinylphenol with LuxO of *Vibrio cholerae*, which forms hydrogen bond interaction with TYR129 and GLN363. **(B)** Interaction of hexahydro-3-(phenylmethyl)-pyrrolo[1,2-*a*]pyrazine-1,4-dione with HapR of *V. cholerae*, forming hydrogen bond with SER165. The *V. cholerae* response regulators are represented as surface (LuxO, gold color; and HapR, blue color), and the small molecules are represented as sticks.

**FIGURE 8:**
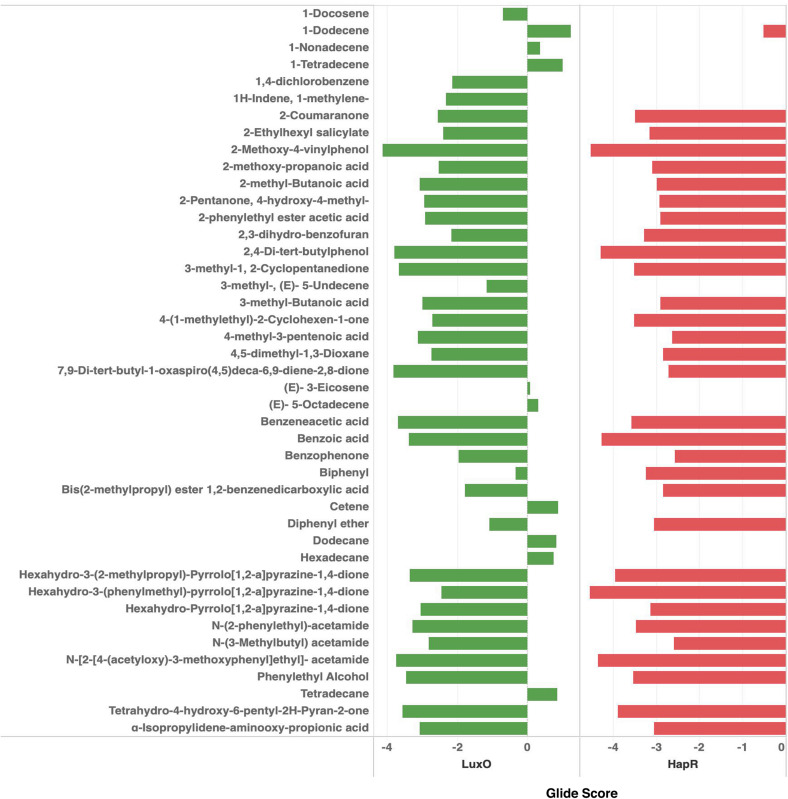
Docking scores of the bioactive compounds identified in the ethyl acetate extract of RMA46 with LuxO and HapR of *Vibrio cholerae*.

*QS global regulators*: LuxO and HapR drive the virulence mechanism including biofilm and CTX production in *V. cholerae* in response to low and high environmental signals. Because an experimentally solved structure of LuxO of *V. cholerae* is not available, we used a 1.6-Å resolution X-ray structure of the LuxO receiver-catalytic domain of *Photobacterium angustum*, which has 69% of sequence identity as a template for modeling *V. cholerae* LuxO (Figure 6). Any gaps observed during core modeling were subjected to loop modeling using Schrödinger PRIME (Jacobson et al., 2004). Then, the modeled protein was subjected to a constrain-based energy minimization using GROMACS. The Ramachandran plot of the modeled protein showed that PHI and PSI angles of the modeled proteins are within the allowed ranges, and the modeled protein was acceptable and hence used for further docking studies. For HapR, an existing X-ray structure of 2.2-Å resolution with putative ligand-binding domains was used. The small molecules identified in the RMA46 ethyl acetate extract identified using GC–MS was docked with the active sites of LuxO and HapR (Figure 7). Compound 2-methoxy-4-vinylphenol demonstrated an increased Glide score (-4.130) and Glide energy (-20.408 kcal/mol) with LuxO protein than did other molecules (Figure 8). The interaction pattern was observed as the ligand forms hydrogen bonding with the most crucial amino acid residues (GLN363 and TYR139) of the receiver-catalytic domain of ATP binding domain of LuxO. Similarly, the ligand, hexahydro-3-(phenylmethyl)-pyrrolo[1,2-*a*]pyrazine-1,4-dione, was found to have the highest affinity with the HapR protein with a Glide score of -4.543 and Glide energy of -30.465 kcal/mol and formed hydrogen bond interaction with SER165 amino acid.

## Discussion

The emergence of antimicrobial resistance among *Vibrio cholerae* is a burden on public health especially in developing countries (Das et al., 2019). An increase in the incidence of multidrug resistance *V. cholerae* could hurt cholera-controlling measures within a community (Mushayabasa and Bhunu, 2012). To subdue the emergence of antibiotic resistance, natural compounds that exert anti-virulence properties have been recognized as an alternative strategy (Narendrakumar et al., 2019a). Marine-derived Actinobacteria are a rich source of secondary metabolites that provide unique carbon scaffolds for the synthesis of advanced therapeutic agents for pathogens (Hassan and Shaikh, 2017). More than 3,000 compounds have been reported from marine bacteria most prevalently from the phylum Actinobacteria, which are known to inhibit drug-resistant bacterial pathogens (Rahman et al., 2010). The density of saprophytic Actinobacteria is high in mangrove soil owing to the detritus ecosystem found throughout the year (Saravanakumar et al., 2016). In this study, Actinobacteria strains were isolated from mangrove soil by various physical and chemical pretreatments by using a wide range of selective isolation media. It was observed that pretreatment of soil did not significantly affect the isolation of Actinobacteria strains. However, the application of starch casein agar and ISP no. 4 medium was most effective in the isolation of the Actinobacteria. The phylum Actinobacteria is one of the largest among the prokaryotes and encompasses many genera that are morphologically and phenotypically diverse.

The preliminary identification using light microscopy and colony morphology showed that the isolates were morphologically diverse, indicating the isolation of various genus. The isolated RMAs were screened using HYR14, a rugose *V. cholerae* O1 Ogawa biofilm-forming strain isolated during an outbreak in India. The strain is resistant to ampicillin, furazolidone, nalidixic acid, streptomycin, ciprofloxacin, erythromycin, and norfloxacin (Chowdhury et al., 2016). Because the rugose phenomenon is stable even after passage into humans and their potential to cause epidemics, the multidrug-resistant HYR14 strain was chosen for the screening the RMAs that can inhibit biofilm formation by *V. cholerae* for the current study (Morris et al., 1996; Chowdhury et al., 2016).

Although marine Actinobacteria account for 46% of the total Actinobacteria density exerting virulence inhibitory potential (Sarveswari and Solomon, 2019), the exploration for anti-virulence agents against *V. cholerae* had been very limited. Previously, culture supernatant of arctic derived rare Actinobacteria, *Nocardiopsis* sp. A731, had been reported to exhibit a biofilm inhibitory activity against *V. cholerae* (Augustine et al., 2012). In our study, anti-biofilm phenotype-based screening of the RMA against HYR14 strain revealed that about 19% of the mangrove-derived isolates could inhibit the formation of *V. cholerae* biofilm. The study reinforces that the mangrove-derived Actinobacteria are a prospective source for anti-infectives against *V. cholerae*. Moreover, the HYR14 cell-free extract inhibits the formation of *V. cholerae* biofilms at a concentration (v/v) lesser than that of the *Nocardiopsis* sp. A731 reported by Augustine et al. (2012). In the same study, 200 μg/ml of *Streptomyces* sp. A745 diethyl ether fraction has been reported to suppress 60% of *V. cholerae* biofilm. Yet in our study, about 50% inhibition was observed at 130 μg/ml of RMA46 ethyl acetate extract. Although eight RMA isolates showed a significant activity against HYR14 biofilms, only the RMA46 strain that showed > 50% biofilm inhibition at least concentration was further subjected to nucleic acid-based identification. The sequence of the mangrove-derived *Micromonospora* sp. RMA46 was found to be closely related to the 16S rRNA sequence of another mangrove isolate *Micromonospora* sp. 171111. As both the strains were isolated from the mangrove region, the sequence similarity could reflect an environment selection among *Micromonospora* strains (Hong et al., 2009). The RMA46 ethyl acetate extract clearly eliminates the formation of HYR14 biofilm than its eradication. Previously, a non-significant impact on eliminating the preformed *V. cholerae* biofilms by natural products was attributed to their potential to affect the initial stages of biofilm formation including motility (Narendrakumar et al., 2019b). Hence, further studies with the *V. cholerae* HYR14 are needed to understand the mechanism of inhibition of biofilm formation.

The signal (autoinducer) strength influences the QS global switches (LuxO and HapR) to enhance the survivability and persistence of the intestinal pathogen, *V. cholerae* (Joelsson et al., 2007). At LSS, LuxO is positively regulated, and its synergistic interaction with the global response regulator, TsrA, enhances the production of *Vibrio* polysaccharide (VPS) TCP and CT to establish biofilm and virulence (Zheng et al., 2010).

When the signal strength is elevated (HSS), HapR is depressed, resulting in the upregulation of hemagglutinin/protease (HapA), facilitating the dispersal of *V. cholerae* cells within the biofilm matrix (Silva et al., 2003; Vance et al., 2003; Benitez and Silva, 2016). Also, HSS is directly proportional to the increased population size of *Vibrio* in an environment, which might lead to nutrient depletion. Interestingly, under nutrient-limited condition, the expression of HapA protease increases owing to the elevated intracellular cAMP pool. The cAMP binds to its allosteric receptor and exerts a positive feedback loop to activate CRP, RpoS, and HapR (Benitez and Silva, 2016). Our transcriptomic analysis revealed that the expression of major virulence genes and the response regulators of *V. cholerae* HYR14 is highly attenuated without affecting their growth in the presence of RMA46 ethyl acetate extract. This overall attenuation of the molecular machinery activated at LSS and HSS might be a combinatorial effect of two or more potential virulence attenuating bioactive molecules present in the RMA46 ethyl acetate extract against *V. cholerae*.

Genetically marine Actinobacteria are equipped to produce about 20 secondary metabolites (Lam, 2006). In this study, 43 secondary metabolites from various classes of chemical compounds have been identified. The RMA46 extract encompasses many chemicals from the class of carboxylic acid, and interestingly our group had previously reported the QS inhibitory effect of synthetically derived pyrazine-2-carboxylic acid on *V. cholerae* (Hema et al., 2017). The derivative of various other compounds reported in RMA46 has been reported to have other biological activities including antibacterial and anti-virulence activities against various other pathogens. This eventually exemplifies the potential of *Micromonospora* sp. RMA46 as a source for chemical structural templates for the development of anti-virulence agents. Docking of the 43 chemical compounds leads to the identification of 2-methoxy-4-vinylphenol and hexahydro-3-(phenylmethyl)-pyrrolo[1,2-*a*]pyrazine-1,4-dione to have the highest interacting capability with LuxO and HapR, respectively. The data corroborate our qRT-PCR data, which were also significant to show a complete blockage of virulence so as to prevent the colonization and dissemination of bacteria to persist infection. A derivative of 2-methoxy-4-vinylphenol has already been reported to have an inhibitory activity against LasIR/RhIIR circuit of *Pseudomonas aeruginosa* (Shah et al., 2019). Interestingly, hexahydro-3-(phenylmethyl)-pyrrolo[1,2-*a*]pyrazine-1,4-dione, also known as cyclo D-phenyalanyl-L-prolyl [Cyclo(Phe-Pro)], has been reported to attenuate the expression of genes associated with virulence in *V. cholerae*. Cyclo(Phe-Pro) inhibits the production of CTX and toxin coregulated pili in O1 El Tor strain by negatively affecting the transcription of genes *tcp*PH in *V. cholerae* (Bina and Bina, 2010). It was later shown that Cyclo(Phe-Pro) enhanced the ToxR-dependent transcription of *leu*O, which in turn repressed the downregulation of the ToxR regulon, altering the production of CT and TCP (Bina et al., 2013). However, the outcomes derived from this research support the bioprospecting of mangrove-derived *RMA* as sources of potential anti-infectives.

## Conclusion

The pathogenesis of *Vibrio cholerae* is controlled via the QS-mediated communication process. The present study provides an insight in to the bioprospecting of mangrove-derived RMA to block QS-mediated response in *V. cholerae*. *Micromonospora* is one of the most productive genera among the phylum Actinobacteria in terms of the production of bioactive compounds. Yet the study of anti-virulence compounds with this genus is very limited. The Indian Ocean encompasses an astonishing source of marine microbial wealth, providing an opportunity to extensively exploit the RMA for the development of anti-virulence agents. The tapping of effective compounds from RMAs is achievable with the rapid development of modern technology in the field of sampling, isolation, and identification strategies that open up novel mechanisms to arrest the development of rapid dissemination of antimicrobial resistance and pathogenesis of human and animal pathogens. To conclude, the identification of the anti-virulence property of *Micromonospora* sp. is another example of Actinobacteria as a treasure trove for pharmaceutically important compounds.

## Data Availability Statement

The datasets presented in this study can be found in online repositories. The names of the repository/repositories and accession number(s) can be found in the article/Supplementary Material.

## Author Contributions

HS conducted all the experiments described in this study. SK supported the acquisition of images. KS designed the computational work. AS and PN designed the study and supervised all the experiments. HS and SK drafted the manuscript and it was reviewed by SK, PN, and AS.

## Conflict of Interest

The authors declare that the research was conducted in the absence of any commercial or financial relationships that could be construed as a potential conflict of interest.

## Footnotes

1 http://www.chemaxon.com

2 www.uniprot.org

3 https://servicesn.mbi.ucla.edu/SAVES
