# Supplementary material for: Exploration of Anti-infectives From Mangrove-Derived Micromonospora sp. RMA46 to Combat Vibrio cholerae Pathogenesis
Source: Front Microbiol. 2020 Jul 10;11:1393. doi: 10.3389/fmicb.2020.01393 (PMC7381277; doi:10.3389/fmicb.2020.01393)
Supplement: Supplementary file 1 [file Table_1.docx]

**Supplementary Material**

**Exploration of anti-infectives from mangrove-derived *Micromonospora sp.* RMA46 to combat *Vibrio cholerae* pathogenesis**

Hema Bhagavathi Sarveswari^1^, Shanthini Kalimuthu^1^, Karthi Shanmugam^1^, Prasanna Neelakantan^2^*and Adline Princy Solomon^1^*

**Table S1:** Sampling site information

| **Properties** | **Description** |
| --- | --- |
| Sample type | Soil |
| Latitude | 10.340406° N |
| Longitude | 79.539833° E |
| Site | An isolated island inhabited by mangroves |
| pH | 7.9 |
| Salinity (PSU) | 30-40% |
| Temperature | 28°C |

**Table S2:** List of media used for selective isolation of Rare Marine Actinobacteria and also for fermentation

| **Media** | **Basal composition and pH** |
| --- | --- |
| ISP Medium No. 1  (Tryptone Yeast Extract broth) | Casein enzymic hydrolysate, 5 g/L; yeast extract, 3 g/L; pH 7.0 + 0.2 |
| ISP Medium No. 2  (Yeast malt Agar) | Peptone, 5 g/L; yeast extract, 3 g/L; malt extract, 3 g/L; dextrose, 10 g/L; agar, 20 g/L; pH 6.2 + 0.2 |
| ISP Medium No. 3  (Oatmeal agar) | Oat meal, 20g/L; trace salts solution, 1 ml; agar, 18 g/L; pH 7.3 + 0.2 |
| ISP Medium No. 4  (Inorganic Salt Starch Agar) | Starch soluble, 10g/L; dipotassium hydrogen phosphate, 1g/L; magnesium sulphate heptahydrate,1g/L; Sodium chloride,1g/L; Ammonium sulphate, 2 g/L; calcium carbonate, 2 g/L; ferrous sulphate heptahydrate, 0.001 g/L; Manganous chloride, heptahydrate, 0.001 g/L; zinc sulphate heptahydrate, 0.001 g/L; agar, 20 g/L; pH7.2 + 0.2 |
| ISP Medium No. 5  (Glycerol Asparagine Agar Base); | L-Asparagine, 1 g/L; Dipotassium phosphate, 1 g/L; trace salts solution, 1 ml/L; Agar, 20 g/L;pH 7.4 +0.2 |
| ISP Medium No. 6  (Peptone Yeast Extract Iron Agar); | Peptone, 15g/L; proteose peptone, 5 g/L; yeast extract, 1g/L; ferric ammonium citrate, 0.5g/L; dipotassium citrate, 1 g/L; sodium phosphate, 0.08g/L and agar, 15g/L; pH 6.7 + 0.2 |
| ISP Medium No. 7  (Tyrosine Agar) | L-asparagine, 1g/L; L-tyrosine, 0.50 g/L; dipotassium phosphate, 0.5g/L; magnesium sulphate∙7H_2_O, 0.5g/L; sodium chloride, 0.5g/L; trace salt solution, 1ml; agar, 20 g/L; pH 7.3 + 0.1 |
| Starch Casein Agar | Starch, 10 g/L; casein powder, 1 g/L; sea water, 37 g/L; agar 15 g/L; pH 7.2 + 0.2 |
| Actinomycete Isolation Agar | : Sodium caseinate, 2 g/L; L-asparagine, 0.100 g/L; sodium propionate, 4 g/L; dipotassium hydrogen phosphate, 0.5 g/L; magnesium sulphate 0.1 g/L; ferrous sulphate, 0.001 g/L; pH 8.1+ 0.2 |
| Humic acid-Vitamin Agar | Humic acid, 1.0 g/L; potassium chloride , 1.7 g/L; sodium phosphate dibasic, 0.5 g/L; magnesium sulphate heptahydrate, 0.5 g/L; calcium carbonate, 0.02 g/L; ferrous sulphate heptahydrate, 0.01 g/L; agar, 15 g/L; and vitamins (vitamin B6, riboflavin, aminobenzoic acid, pantothenic acid, inositol, thiamine, nicotinic acid (0.5mg) and biotin (0.25 mg); pH7.2 + 0.2. |

**Table S3:** Culture morphology of RMA isolate from mangrove soil

| Isolate No. | Colony Colour | Aerial Mycelium | Substrate Mycelium | Mycelial Colour | Reverse Pigmentation | Soluble Pigment |
| --- | --- | --- | --- | --- | --- | --- |
| 1 | Orange | Present | Present | Orange | Orange | Nil |
| 2 | Brown | Present | Present | Brownish Orange | Brown | Nil |
| 3 | Orange | Present | Present | Orange | Orange | Nil |
| 4 | Black | Present | Absent | Brown | Black | Yes |
| 5 | Orange | Present | Present | Orange | Orange | Nil |
| 6 | Brownish Orange | Present | Present | Brownish orange | Brownish orange | Nil |
| 7 | Brownish Orange | Present | Present | Brownish Orange | Brownish Orange | Nil |
| 8 | Orange | Present | Absent | Orange | Orange | Nil |
| 9 | Orangish Brown | Present | Present | Brown | Brown | Nil |
| 10 | Orange | Absent | Absent | Orange | Orange | Nil |
| 11 | Orange | Present | Present | Orange | Orange | Nil |
| 12 | Orange | Present | Present | Orange | Orange | Nil |
| 13 | Orangish Brown | Present | Present | Orangish brown | Orangish Brown | Nil |
| 14 | Yellow | Absent | Absent | Yellow | Yellow | Nil |
| 15 | Pink | Present | Present | Pink | Pink | Nil |
| 16 | White | Present | Present | White | White | Nil |
| 17 | Bright Orange | Present | Present | Bright Orange | Orange | Nil |
| 18 | Black | Present | Present | Black | Black | Nil |
| 19 | Golden Yellow | Absent | Absent | Golden Yellow | Golden Yellow | Nil |
| 21 | Orange | Absent | Absent | Pinkish Orange | Pinkish Orange | Nil |
| 22 | Orange | Absent | Absent | Orange | Orange | Nil |
| 23 | Yellow | Absent | Absent | Yellow | Yellow | Nil |
| 24 | Black | Present | Present | Black | Black | Nil |
| 26 | Yellow | Absent | Absent | Yellow | Yellow | Nil |
| 28 | Creamy White | Absent | Absent | White | White | Nil |
| 29 | Brown | Present | Present | Brown | Brown | Nil |
| 30 | Lemon Yellow | Absent | Absent | Lemon Yellow | Lemon Yellow | Nil |
| 31 | Orangish Brown | Present | Present | Orangish Brown | Orange Brown | Nil |
| 32 | Creamy White | Absent | Absent | Creamy White | Creamy White | Nil |
| 33 | White powdery | Absent | Absent | White | White | Nil |
| 35 | Dark brown | Present | Present | Dark brown | Dark brown | Nil |
| 37 | Orange | Present | Present | Orange | Orange | Nil |
| 38 | Ivory | Absent | Absent | Ivory | Ivory | Nil |
| 39 | Yellow | Absent | Absent | Yellow | Yellow | Nil |
| 40 | White | Present | Present | White | White | NIl |
| 41 | White | Present | Present | White | White | NIl |
| 42 | Orange | Present | Present | Orange | Orange | Nil |
| 44 | Greyish black | Present | Present | Black | Black | Yes |
| 45 | Orange | Present | Present | Black | Orange | Nil |
| 46 | Orange | Present | Present | Orange | Orange | Nil |
| 49 | Yellow | Present | Present | Yellow | Yellow | Nil |
| 50 | Orange | Present | Present | Orange | Orange | Nil |

**Table S4.** Primer sequence used for quantitative PCR

| **S.No** | **Gene** | **Gene**  **Function** | **Forward primer sequence**  **(5’ – 3’)** | **Reverse primer sequence**  **(3’ – 5’)** | **Reference** |
| --- | --- | --- | --- | --- | --- |
|  | *ct* | Encodes for cholera toxin | TATGCCAAGAGGACAGAGTGAG | AACATATCCATCATCGTGCCTAAC | Sarkar et al., 2002 |
|  | *tcp* | Encodes for toxin co-regulated pili- Adhesion | CGTTGGCGGTCAGTCTTG | CGGGCTTTCTTCTTGTTCG | Sarkar et al., 2002 |
|  | *hapA* | Protease -Dissemination of *V. cholerae* from host intestine | ACGGTACAGTTGCCGAATGG | GCTGGCTTTCAATGTCAGGG | Silva et al., 2006 |
|  | *hapR* | Global response regulator | CCAACTTCTTGACCGATCAC | GGTGGAAACAAACAGTGGCC | Silva and Benitez., 2004 |
|  | *qrr-2* | Small regulator RNA- along with Hpq binds to hapR-repression of HapR | GGTGACCCTTGTTAAGCCGA | CTATTCACTTCAACGTCAGTTGGC | Hema et al., 2017 |
|  | *qrr-4* | Small regulator RNA- along with Hpq binds to hapR- repression of HapR | TGACCCTTCTAAGCCGAGGG | GAACAATGGTGTTCACTTCAACG | Hema et al., 2017 |
|  | *rec A* | DNA cell repair and cell division | ATTGAAGGCGAAATGGGCGA | TACACATACAGTTGGATTGC | Lipp et al., 2003 |

**Figure S1. Effect of various concentrations of cell free extract on growth of *V. cholerae* HYR14**


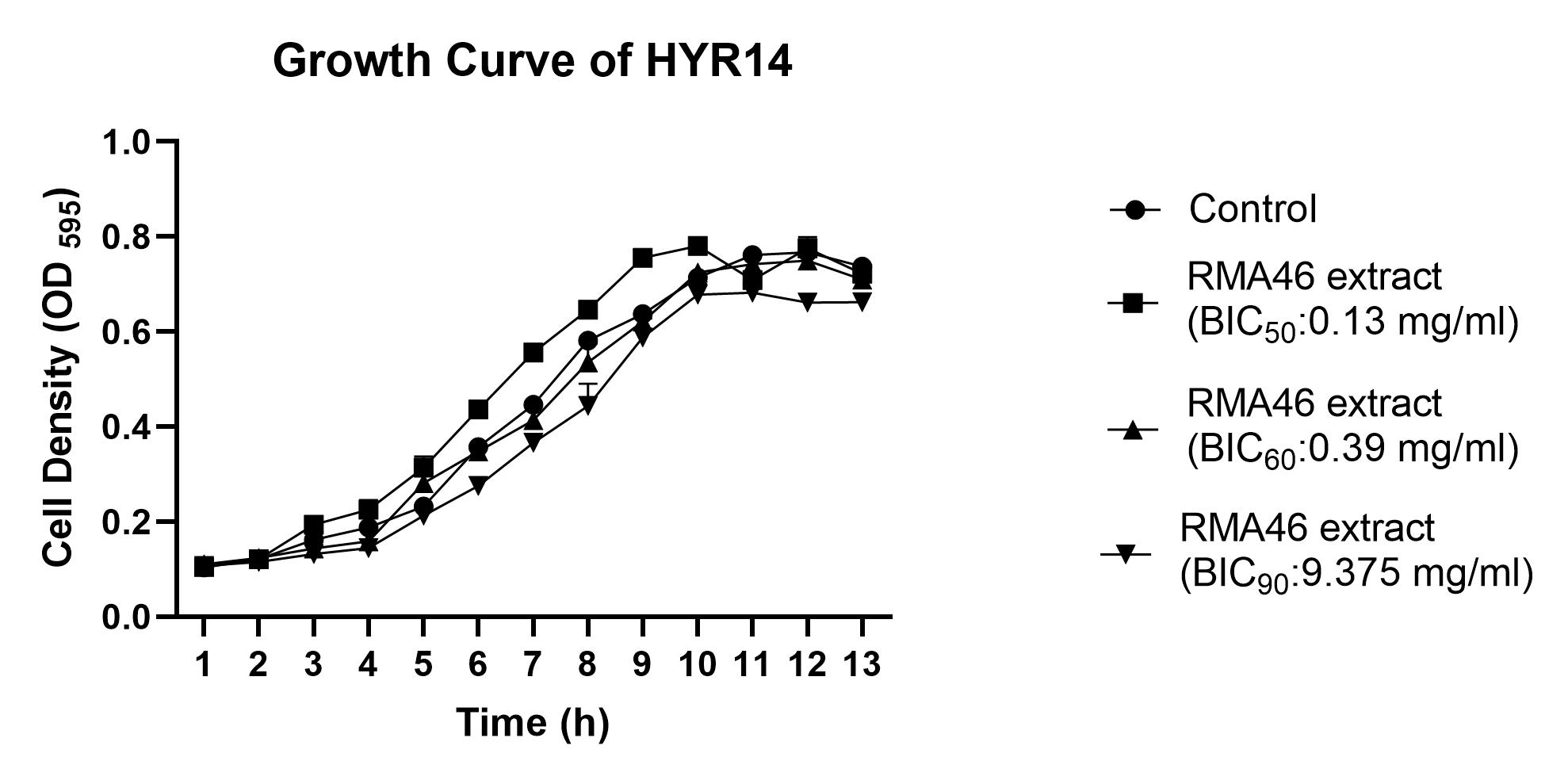


**Figure S2. Neighbor-joining tree-based relationship analysis of *Micromonospora* sp. RMA46**

**
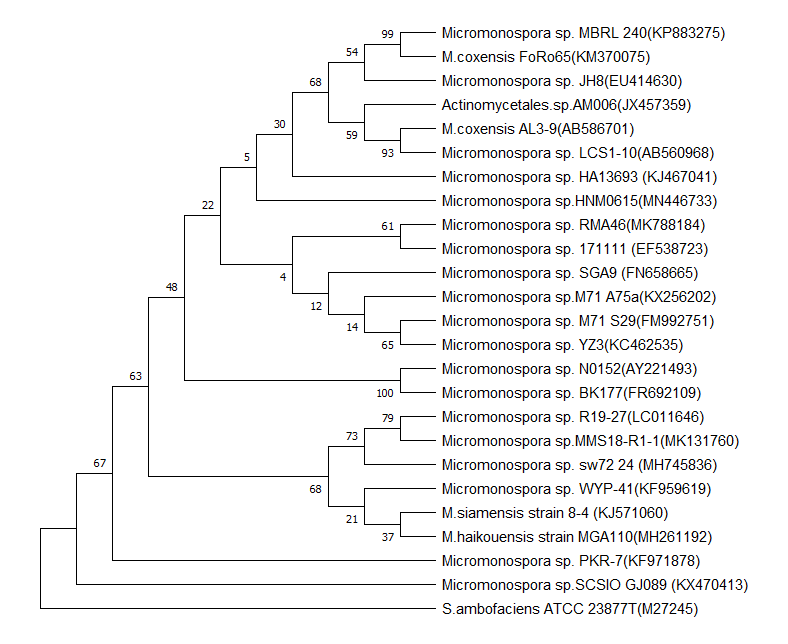
**

**Figure S3. Total ion chromatography of *Micromonospora* sp. RMA46 ethyl acetate extract**

**
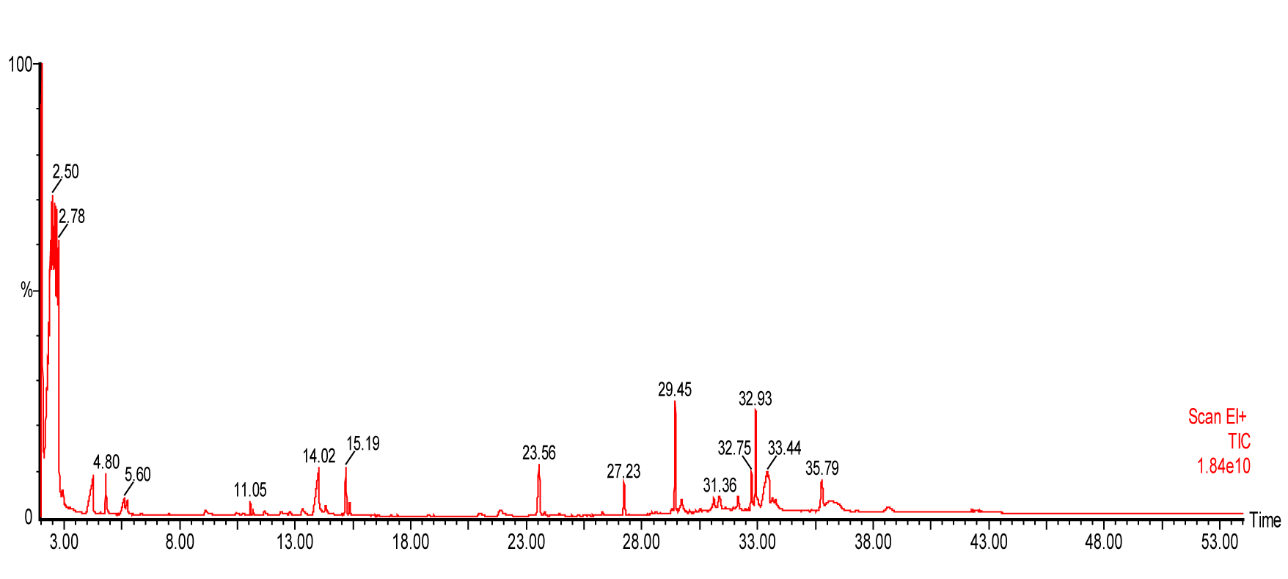
**
